# Health Technology Assessment and Cardiology: A Review of the Present and Future of Innovation

**DOI:** 10.3390/jmahp13020030

**Published:** 2025-06-09

**Authors:** Ruben Casado-Arroyo, Lucia Osoro

**Affiliations:** Department of Cardiology, H.U.B.-Hôpital Erasme, Université Libre de Bruxelles, 1070 Brussels, Belgium

**Keywords:** patient-centric solutions, health technology assessment, cardiology, innovation, medical devices

## Abstract

**Background and Objective:** Innovation is a key enabler of patient-centered care in cardiology, with new medical devices and digital health technologies offering the potential to improve outcomes and efficiency. However, the evaluation of these innovations poses challenges for clinicians, regulators, and procurement stakeholders, particularly within the complex European healthcare landscape. This review aims to explore the current state of health technology assessment (HTA) for cardiology-related medical devices in Europe, offering a clinical perspective. **Material and Methods:** Three independent scoping reviews were conducted following the PRISMA-ScR guidelines. Keywords included “innovation”, “health technology assessment”, and “cardiology”. The search was supplemented by the relevant literature on European HTA policies, regulatory directives, and emerging technologies. **Results:** The review identified three central themes: (1) the evolving role of clinicians in HTA processes, (2) the integration of innovative technologies such as digital tools and artificial intelligence within HTA frameworks, and (3) the considerable variation in HTA practices and policies across EU member states. **Conclusions:** HTA in Europe is undergoing a transformation, with increasing emphasis on interdisciplinary collaboration and frameworks that support innovation. While the goal of harmonization across the EU remains a work in progress, new regulatory efforts, such as the HTA Regulation (HTAR), offer promising avenues for aligning clinical practice with evidence-based assessment and reimbursement decisions.

## 1. Introduction

Over recent decades, life expectancy has significantly increased, with projections indicating a 35% rise in the population over the age of 50 and a 300% increase in those over 85 by the year 2050. While this demographic shift reflects advancements in healthcare and living conditions, it also correlates with a growing burden of age-related conditions—particularly cardiovascular diseases (CVD), which are predominantly degenerative in nature [1].

In Europe, these trends mirror those observed in the United States, where it is estimated that 40% of the population will experience some form of CVD by 2030. CVD remains the leading cause of mortality across the EU, accounting for approximately 4.3 million deaths annually and costing the region an estimated EUR 200 billion per year [1].

Technological innovation in cardiology has played a pivotal role in reducing CVD-related mortality. Between 1980 and 2000, it is estimated that 25% of the decline in age-adjusted death rates from CVD can be attributed to the introduction of novel therapies, medical devices, and procedural techniques [2,3]. Among these, cardiovascular medical devices and digital health tools are expected to be key drivers of future progress, contributing to the shift toward value-based, patient-centered care [3].

To ensure the effective integration of such innovations into clinical practice and public health systems, robust health technology assessment (HTA) mechanisms are essential. HTA is a multidisciplinary process that evaluates the value of health interventions throughout their lifecycle with the goal of supporting evidence-informed decision-making and promoting equitable, high-quality healthcare delivery [4]. Cross-sector collaboration among clinicians, patients, payers, and public institutions is fundamental to this process. Notably, the European Network for Health Technology Assessment (EUnetHTA) facilitated such coordination before its renewal under the EUnetHTA21 initiative [5,6].

In preparation for the implementation of the new EU Regulation on Health Technology Assessment (HTAR, Regulation 2021/2282), the European Medicines Agency (EMA) and EUnetHTA21 have been collaborating to align assessment methodologies and procedural standards across the region [7]. The HTAR, which takes effect in January 2025, introduces a harmonized framework for evaluating high-risk medical devices, enabling shared clinical assessments and a more streamlined regulatory process [8,9].

Given that approximately 50% of all class III high-risk medical devices are used in cardiology, the implications of this regulation are particularly relevant for cardiovascular innovation [1]. Initiatives such as the European Society of Cardiology (ESC) led evaluation of wearable cardioverter-defibrillators exemplify efforts to align clinical guidelines with HTA outcomes. These efforts underscore the value of involving cardiologists in the generation and review of clinical evidence, as well as in regulatory and reimbursement processes [5].

Despite these advancements, digital health tools (DHTs) and artificial intelligence (AI)-based technologies continue to present unique challenges for HTA. Unlike traditional devices, DHTs evolve rapidly, with frequent software updates and shorter innovation cycles, which current HTA models are often ill-equipped to accommodate [10,11].

In this dynamic landscape, stakeholders face increasing pressure to balance innovation with regulatory oversight, economic evaluation, and timely market access. This review explores the critical role of cardiologists in shaping HTA practices, the importance of cross-sector collaboration, and the need to adapt existing frameworks to address digital innovation and geographical heterogeneity in HTA implementation across Europe [10].

## 2. Methods

The development of this manuscript was supported by 23 publications, and it was performed in two phases. Phase one was conducted by searching papers on the domain of interest: HTA of cardiology MDs in Europe, from relevant organizations such as the ESC and the European Commission (EC).

**Figure 1:**
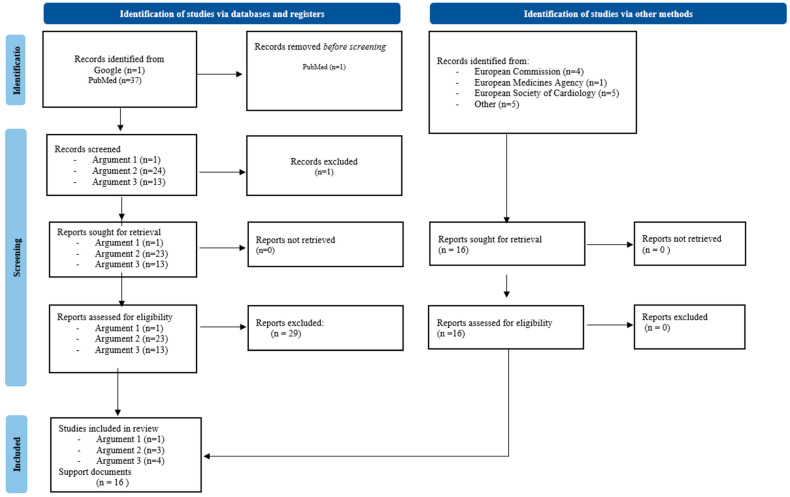
PRISMA flow.

For the second phase, the authors of the paper identified three main arguments of interest: the role of cardiologists and potential partnerships, the challenges for new devices and DHTs, and the HTA diversity across Europe. For this purpose, we performed three independent searches following the revised 2020 Preferred Reporting Items for Systematic Reviews and Meta-Analyses (PRISMA) research flow. We searched for information in PubMed and Google using the same filters for the three arguments: “Free full text”, published from 1 January 2019 to 31 December 2024, and with English as document language (Figure 1).

For the first argument, “the role of cardiologists in HTA”, we used the following search keywords: “cardiologists”, “HTA”, “innovation”, and “medical device”. We did not obtain any paper supporting these topics in PubMed. Therefore, we performed a hand search using Google with the statement: “The role of cardiologists in Health Technology Assessment”. Out of the 186 entries obtained, only one refers to the topic of interest, and it was published during the last 5 years and was free to access (Table 1).

For the second argument, “HTA of innovative solutions” search, we used the following keywords: “HTA”, “innovation”, “cardiology”. We obtained 24 publications. From these 24 publications, we excluded an erratum document from the same paper and screened 23 publications. For the screening phase, the inclusion criteria considered were publications on health technology assessment (*n* = 19), cardiology (*n* = 15), and medical devices, leaving three publications meeting both conditions for retrieval (Table 2).

For the third argument, “HTA and geographical disparities across Europe”, we used the keywords: “HTA”, “Europe”, and “Medical Devices”. The result was 13 publications. From these 13 publications, there were no exclusions, so we screened them all. For the screening phase, the inclusion criteria considered were publications on health technology assessment (*n* = 8), cardiology (*n* = 1), OR medical devices (*n* = 5). For retrieval, we considered those that met two out of three criteria (Table 3).

The additional literature used to support the development of this manuscript includes documents from experts within the ESC, documents from the European Commission, authors that have recently discussed relevant information on HTA new technologies such as AI or clinical decision support systems (CDSS), documents on evidence generation and evaluation of cardiology devices.

## 3. Results

During the three independent reviews, the authors attested that there is barely any documentation on the involvement of cardiologists during health technology assessment, with only one valid entry out of the 186 entries provided by the search engine.

Regarding publication type for arguments 2 and 3, the authors found that 62.5% of the papers analyzed the topic of interest, while the rest, 37.5%, were based on surveys and interviews.

The authors identified three crucial topics: the role of cardiovascular healthcare providers and their involvement in the HTA processes. The impact of innovative solutions in cardiology, such as AI, and how they converge in the healthcare ecosystem to be reimbursed for use. Finally, the challenges and differences in the HTA processes across the European countries that directly impact the adoption of innovative solutions in the framework of the cardiology department.

### 3.1. Role and Necessary Involvement of Cardiologists in HTA

Understanding the role of clinicians in the innovation and HTA landscape requires first clarifying their professional function. A clinician is defined as “an individual who utilizes a recognized scientific knowledge base and has the authority to direct the delivery of personal health services to a patient”. Within the HTA context, clinicians are not only end-users of medical innovations but also vital contributors to their development and evaluation [11].

Historically, clinicians have played a catalytic role in driving the development of novel medical devices (MDs). Their involvement has often been concentrated in the clinical validation phase; assessing safety, efficacy, and comparative performance. However, their participation has largely been peripheral to the core HTA process. Clinicians are uniquely positioned to assess the real-world clinical need for new technologies, making them indispensable to early-stage HTA activities such as horizon scanning, defining clinical endpoints, and contributing to guideline development [11].

In cardiology, where technologies such as implantable cardiac electronic devices (ICEDs) are frequently introduced, rigorous standards are required. These devices typically demand high-quality clinical evidence, often generated through randomized controlled trials (RCTs), to demonstrate efficacy and justify reimbursement. Despite the availability of such evidence, numerous barriers hinder the integration of these technologies into routine care. Discrepancies in reimbursement policies, misalignment between stakeholders, and conflicting priorities among clinicians, administrators, and payers all contribute to fragmented adoption pathways [10].

The emergence of digital health tools (DHTs) further complicates this landscape. These technologies, including AI-based diagnostics, remote monitoring apps, and clinical decision support systems, are now regulated as medical devices under EU law. However, the frameworks for assessing their clinical value, utility, and cost-effectiveness remain underdeveloped. Unlike traditional devices, DHTs often have shorter development lifecycles, frequent software updates, and a need for continuous validation. These characteristics do not align well with conventional HTA models, which are typically more static and evidence-intensive [10].

Nevertheless, there is a growing consensus around the value of harmonized clinical practice guidelines, such as those issued by the ESC, in guiding reimbursement and HTA decisions. These guidelines serve as a bridge between clinical relevance and policy-making, supporting consistent evaluation criteria across jurisdictions [19].

Given this context, there are significant opportunities to strengthen collaboration among clinical societies, national HTA bodies, EU regulatory institutions, and industry stakeholders. Such partnerships can enhance the robustness of clinical evidence generation, ensure alignment with real-world practice, and foster dialog that balances innovation with health system sustainability. Cardiologists, in particular, are well-placed to lead this charge by contributing clinical insights that shape HTA frameworks from both scientific and patient-centered perspectives [19].

### 3.2. HTA of Innovative Solutions

Medical devices and DHTs play a critical role in enhancing access, quality, and efficiency in healthcare, and are instrumental in the transition toward value-based care models. By supporting patient-centered approaches and enabling more precise diagnostics and interventions, these innovations help deliver improved health outcomes across a broad range of clinical contexts. However, the evaluation of such technologies through HTA frameworks presents several persistent challenges [3,12].

Unlike pharmaceuticals, medical devices are often characterized by steep learning curves that depend heavily on operator expertise and real-world usage patterns. They are also subject to frequent technical modifications and iterative development, which complicates the design and execution of robust RCTs, the traditional gold standard for generating clinical evidence. These features introduce variability and limit the generalizability of trial results, thereby reducing the ability of standard HTA models to fully capture the clinical and economic value of these technologies [12].

Nevertheless, HTA can be strategically applied in the early stages of technology development. Early HTA enables stakeholders to forecast clinical impact and cost-effectiveness even before full-scale market entry, thereby informing investment decisions, guiding research prioritization, and improving budget allocation. This approach is particularly valuable for high-risk or high-cost innovations where uncertainty can delay adoption [13].

Innovative digital technologies, especially those based on AI, face additional barriers to adoption. Despite their promise for improving clinical decision-making and efficiency, these tools often struggle to move beyond performance metrics into demonstrable clinical utility. Many AI-based solutions lack the longitudinal or real-world evidence needed for formal HTA, and current regulatory pathways are not yet fully adapted to address their unique life cycles and risk profiles [14].

As a result, there is a growing need to adapt existing HTA frameworks, or develop new ones, that can accommodate the dynamic nature of digital health tools. These frameworks should incorporate flexible, iterative models of evidence generation, including real-world data, user feedback, and staged economic analysis, to ensure that the value of innovation is accurately captured over time [20,21].

In response to these challenges, some stakeholders have begun exploring alternative reimbursement models inspired by value-based arrangements long used in the pharmaceutical sector. In particular, “outcomes-based contracts” and “performance-based agreements” are emerging in the medical device space. These models link payment to the achievement of specific clinical outcomes, shifting the focus from upfront costs to real-world effectiveness and aligning incentives across manufacturers, providers, and payers [14].

### 3.3. HTA and Geographical Disparities Across Europe

Europe presents a diverse and fragmented healthcare environment, where existing payment and delivery models often fail to address critical challenges such as aging populations and the shift toward value-based care. Traditional systems, still oriented around procedural volume rather than patient outcomes, are increasingly misaligned with health policy ambitions that prioritize quality, sustainability, and integration [3].

Although CE marking harmonizes market access at the European level, HTA requirements vary significantly between countries. These differences include assessment timing (pre- vs. post-market), product categories, payer systems, and jurisdictional responsibilities. As a result, manufacturers face inconsistent market access conditions, and in many cases, national HTA bodies assess a medical device only after it has been introduced to the market [15,16].

Variations in HTA methodology may stem from differences in clinical pathways, population health profiles, and national healthcare priorities. These discrepancies lead to divergent standards and rules, creating a patchwork of availability for new treatments across Europe. Despite these differences, comparative effectiveness remains the preferred form of clinical evidence for HTA decisions, especially when evaluating new technologies against the current standard of care [10,17,18].

Evidence requirements themselves are not consistently applied across the EU. A recent analysis showed that only 76% of HTA reports in the EU include RCT data, while 24% rely on real-world evidence (RWE). Yet, no common guidelines exist to standardize the use of RWE among developers and HTA agencies [18]. To address these inconsistencies, the EU introduced a new framework under the HTAR, which became active on 12 January 2025. This regulation aims to improve access to innovation by facilitating joint assessments and enabling broader, faster evaluation of health technologies [22].

However, the HTAR does not prescribe specific assessment methodologies, raising questions about the balance between harmonization and methodological flexibility. While the EUnetHTA model eliminated value judgments from Joint Clinical Assessments (JCAs), countries retain autonomy during national adaptation phases. This leaves whether value judgments should be fully integrated into JCAs or deferred to country-specific evaluations unresolved [23].

The ESC through its Cardiovascular Round Table (CRT), has highlighted several cases where misalignment between clinical need and reimbursement has slowed the adoption of validated technologies. One prominent example is transcatheter aortic valve implantation (TAVI), a minimally invasive intervention shown to be safer than surgical aortic valve replacement (SAVR). Despite support from ESC guidelines, TAVI reimbursement has taken up to six years in some countries post-CE marking. In contrast, Germany implemented an innovation fund that allowed access to TAVI within one year, demonstrating how national mechanisms can expedite or delay access, even in cases of strong clinical evidence. TAVI’s cost (EUR 33,000) versus SAVR (EUR 22,000) remains a factor, but its clinical advantages have been widely recognized [1].

Another example is percutaneous mitral valve repair (PMVR) using the MitraClip™ device, indicated for high-risk patients with moderate to severe mitral regurgitation. Despite CE marking in 2008 and inclusion in ESC guidelines since 2012, the procedure is still not widely reimbursed across Europe. Numerous randomized trials have confirmed its non-inferiority to open surgery, and the technique has demonstrated shorter hospital stays, better recovery, and reduced need for post-acute care. Yet, access remains inconsistent [1].

Similarly, fractional flow reserve (FFR), a diagnostic tool for assessing the severity of coronary artery disease, has become a global gold standard, supported by clinical guidelines from the ESC, American College of Cardiology and American Heart Association. Studies show that FFR can reduce the risk of death, myocardial infarction, and revascularization by nearly 30%, with an 86% reduction in unplanned hospitalizations for urgent procedures. Approved in 1997 and backed by compelling data, FFR is reimbursed in countries such as Germany and the UK, but not in others, including Italy and Switzerland. This inconsistency continues to restrict access to proven, cost-effective technologies [1].

**Figure 2:**
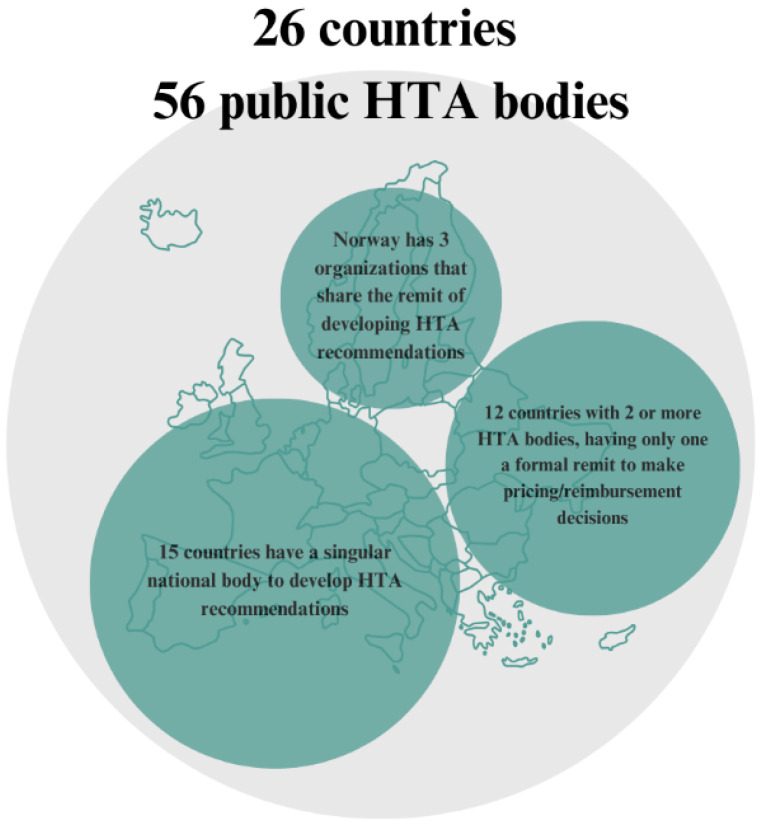
Diversity of public HTA bodies across EU countries.

In terms of structural capacity, 25 EU member states and Norway have national HTA systems in place. Across these 26 countries, 56 organizations identified themselves as HTA bodies. Among them, 15 countries have a single national body producing HTA recommendations, while 12 countries operate multiple organizations involved in HTA production. Norway, for example, has three such entities, with one assuming leadership at a given time (Figure 2). This diversity in the structural capacity exemplifies the heterogenity of the European territory [22].

## 4. Limitations

The research of papers was performed by a single reviewer supervised by a senior researcher. Therefore, there is a risk of some bias related to the classification and scrutiny of papers.

## 5. Discussion

Based on the evidence presented in this manuscript, the new HTAR presents a valuable opportunity to strengthen collaboration across the European Union. The early involvement of cardiologists and professional societies in HTA processes could significantly enhance the relevance and quality of clinical evidence, thereby streamlining evaluations and improving decision-making. However, this also raises important questions regarding the appropriate timing, scope, and extent of clinician engagement, particularly in the assessment of digital technologies.

The evaluation of innovation continues to face considerable challenges. Uncertainty persists about when emerging technologies should be assessed, especially those still under development, and how existing HTA frameworks can adapt to digital health tools characterized by shorter life cycles and rapid iteration. At the same time, the medical device sector is increasingly adopting performance-based and value-driven reimbursement models, mirroring strategies long utilized in the pharmaceutical industry. These new approaches present new opportunities but also challenges to the existing healthcare systems.

With the implementation of the HTAR, the EU seeks to harmonize HTA practices, reduce administrative burdens for manufacturers, and support more equitable access to innovation. Nevertheless, achieving this goal requires a careful balance between methodological standardization and contextual flexibility. The regulation must avoid over-prescriptive approaches while still allowing for national adaptations that account for the diverse healthcare systems across Europe. How this balance will be achieved in practice remains an open and important question.

## 6. Conclusions

The evolution of the practice of cardiology heavily relies on technology. Therefore, HTA is a key pillar to the introduction of innovation which contributes to improving the value delivered to the patients. Standardizing HTA frameworks across Europe is crucial to benefit both users and manufacturers in systems that are moving towards value creation and outcome improvement. With new methodologies to evaluate clinical evidence, medical societies have the opportunity to support the alignment between reimbursement and clinical guidelines, shortening the time to assess innovative medical devices. Moreover, the new HTAR has the potential of creating frameworks across Europe facilitating the process for manufacturers and developers. Nevertheless, future research will be needed in order to evaluate the integration of DHTs in the HTAR processes.

## Figures and Tables

**Table 1:** Search results for argument 1: role of cardiologists in HTA.

Reference	Database	Year	Author(s)	Title
[11]	Google	2019	Smith et al.	Defining the clinician’s role in early health technology assessment during medical device innovation—a systematic review

**Table 2:** Search results for argument 2: HTA in AI and innovative solutions.

Reference	Database	Year	Author(s)	Title	HTA Related? Y/N	Cardiology? Y/N	Medical Devices/DHTs
[12]	PubMed	2020	Inoue et al.	Cost-Effectiveness of Transcatheter Aortic Valve Implantation Using a Balloon-Expandable Valve in Japan: Experience From the Japanese Pilot Health Technology Assessment	Y	Y	Y
[13]	PubMed	2019	Wenker et al.	MRI-guided pulmonary vein isolation for atrial fibrillation: what is good enough? An early health technology assessment	Y	Y	Y
[14]	PubMed	2022	Boriani et al.	Performance-based risk-sharing arrangements for devices and procedures in cardiac electrophysiology: an innovative perspective	Y	Y	Y

**Table 3:** Search results for argument 3: HTA and geographical disparities across Europe.

Reference	Database	Year	Author(s)	Title	HTA Related? Y/N	Cardiology? Y/N	Medical Devices/DHTs
[15]	PubMed	2021	Blankart et al.	Regulatory and HTA early dialogues in medical devices	Y	Y	Y
[16]	PubMed	2021	Federici et al.	Coverage with evidence development schemes for medical devices in Europe: characteristics and challenges	Y	N	Y
[17]	PubMed	2022	Klein et al.	Real-world evidence in health technology assessment of high-risk medical devices: Fit for purpose?	Y	N	Y
[18]	PubMed	2020	Tarricone et al.	Lifecycle evidence requirements for high-risk implantable medical devices: a European perspective	Y	N	Y

## Data Availability

No new data were created or analyzed in this study.
